# Synthetic polymers promote cell aggregation and accelerate cathode-associated layer formation in *Kyrpidia spormannii* EA-1

**DOI:** 10.1016/j.bioflm.2026.100399

**Published:** 2026-09-15

**Authors:** Leonie Rominger, Amit Deb, Christian Jonas Lapp, Francisco (Paco) Fernandez-Trillo, Johannes Gescher

**Affiliations:** a Institute of Technical Microbiology, Hamburg University of Technology (TUHH), Kasernenstraße 12 (F), Hamburg, 21073, Germany; b BioNanoChem Lab, Centro Interdisciplinar de Química y Biología (CICA), and Facultade de Ciencias, Universidade da Coruña, A Coruña, 15071, Spain; c School of Chemistry, University of Birmingham, United Kingdom

**Keywords:** Aggregate formation, Microbial electrosynthesis, *Kyrpidia spormannii*, In-situ screening of polymers, Bioelectrochemical systems, Cathode biofilm

## Abstract

Microbial electrosynthesis using *Kyrpidia spormannii* EA-1 represents a promising approach for sustainable biotechnology, yet the initial biofilm formation on cathode surfaces remains a bottleneck limiting process efficiency. In this study, we investigated the potential of synthetic polymers to accelerate cell aggregation and cathode colonization by this microorganism in bioelectrochemical systems. Polymers were prepared through conjugation, in aqueous conditions, of a poly(hydrazide) (pAcU) with twelve different aldehydes including aromatic, heterocyclic, and aliphatic aldehydes. Crystal violet staining identified four promising polymer-aldehyde conjugates (with 2-naphthaldehyde, 1H-imidazole-4-carbaldehyde, uracil-5-carbaldehyde, and hexanal), one of which significantly increased crystal-violet-retained surface-attached biomass compared to its aldehyde-only control. Growth kinetics revealed that these polymer-aldehyde conjugates initially reduced apparent planktonic growth rates, which may reflect stress responses, reduced viability, or a shift toward an aggregated, surface-associated lifestyle. Live/dead staining demonstrated that cells became entrapped within aggregates, with viability depending on the specific aldehyde used. Transcriptomic analysis revealed upregulation of sporulation genes, toxin-antitoxin systems, and stress response pathways, alongside downregulation of metabolic transporters and carbon metabolism genes, a response compatible with**,** but not specific to, a biofilm-associated lifestyle. In a bioelectrochemical reactor, addition of the polymer-2-naphthaldehyde conjugate accelerated cathode coverage, with a cathode-associated layer reaching over 1 mm in height within seven days, compared to 0.17 mm in the biotic control and 0.24 mm in an abiotic polymer-only control, indicating a substantial contribution of both cellular aggregation and polymer deposition to layer formation. These findings indicate that synthetic polymer-aldehyde-conjugates represent a promising strategy to accelerate cathode colonization during the start-up phase of microbial electrosynthesis processes with *K. spormannii*.

## 1 Introduction

Oxic microbial electrosynthesis (oMES) has emerged as a promising technology for sustainable production of chemicals and fuels from CO_2_ using electroautotrophic microorganisms [1], [2], [3], [4]. In these systems, microorganisms form biofilms on cathode surfaces where they accept electrons to drive CO_2_ fixation, offering an attractive route for converting renewable electricity into valuable biochemicals [5]. Unlike conventional anoxic microbial electrosynthesis that relies on acetogens and methanogens producing mainly acetate or methane, oMES harnesses aerobic hydrogen-oxidizing bacteria (Knallgas bacteria) that can produce more complex products including biomass suitable for food and feed applications [1].

Efficient oMES requires close proximity between electrode and biocatalyst as well as high cell numbers to raise the geometric CO_2_-conversion rate, which is why cathodic biofilms are the configuration of choice not only in oMES but throughout most MES systems [6]. A dense biofilm can act as a functionalization of the electrode surface that lowers the overpotential for proton reduction, both by continuously depleting the hydrogen formed at the cathode and by locally concentrating metal species that act as abiotic catalysts, resulting in a lower energy demand for product formation than in gas fermentation [7]. Because the low aqueous solubility of hydrogen otherwise limits attainable space-time yields and forces energy-intensive mixing for uniform bubble dispersion, a cathode-associated biofilm circumvents this mass-transfer bottleneck by consuming hydrogen directly as it diffuses through the cell layers, before it dissolves or degasses [8]. Beyond these transport and energetic benefits, surface-attached growth retains biomass on the electrode and thereby decouples cell retention from the hydraulic retention time, preventing washout in continuously operated reactors and sustaining the high cell densities required for high volumetric productivity [9]. Accordingly, biofilm-driven microbial electrosynthesis has been reported to outperform suspended-cell configurations by several orders of magnitude for organisms that form robust electrode biofilms [9]. Certainly, some organisms for MES applications might not be good biofilm formers per se and here it might still be best to work with a planktonic system [10] or as shown in this study advance biofilm capabilities synthetically. Finally, in oMES the biofilm contributes to process stability, as it depletes dissolved oxygen before it reaches the cathode, thereby helping to maintain high Faradaic efficiency, and buffers cells against local physicochemical gradients. Nevertheless, at the same time, biofilms remain subject to internal mass-transfer limitations, so that an excessively thick or dense layer is not inherently beneficial.

The cathode material is a key determinant of microbial colonization and cathodic performance, as its surface chemistry, morphology, conductivity and biocompatibility govern initial cell attachment, biofilm establishment and the efficiency of extracellular electron transfer [11]. Moreover, material characteristics and architecture of the electrodes when transferred to three-dimensional flow through electrodes largely determine reactor performance [12]. Carbon-based electrodes, and graphite in particular, are among the most widely used cathode materials in microbial electrosynthesis owing to their biocompatibility, chemical stability, conductivity and low cost. Accordingly, graphite cathodes were used throughout the present study.

Among the microorganisms capable of electroautotrophic growth, the thermophilic bacterium *Kyrpidia spormannii* EA-1 has attracted considerable attention due to its ability to form robust cathodic biofilms and its potential for producing biomass from CO_2_ and electricity as well as polyhydroxyalkanoates (PHAs) [13], [14], [15]. This thermoacidophilic organism, isolated from hydrothermal systems at São Miguel Island (Azores, Portugal), grows optimally at 60 °C and pH 3–4 on cathodes poised to potentials around −500 mV vs. SHE [13], [16]. Remarkably, *K. spormannii* forms biofilms exceeding 100 μm thickness on graphite electrodes which is substantially thicker than those achieved by the mesophilic model organism *Cupriavidus necator* [14], [17], [18]. However, biofilm thickness is not in itself a measure of biofilm quality or performance. The parameter that ultimately governs reaction performance is the quantity of metabolically active, electrode-connected biomass and its associated rate of electron uptake and CO_2_ fixation. Greater thickness is beneficial only where it corresponds to additional active biomass close to the electrode, and up to the point at which mass-transfer limitations for hydrogen, oxygen, CO_2_ and protons begin to restrict the activity of the innermost cell layers. This activity of cathodic biofilms could be judged for instance by electron uptake activity. A recent study revealed that cathodic growth even under oxic conditions can be highly energy efficient, as complete coverage of the electrode led to a maximum of almost 100% biological electron uptake with no abiotic electron losses, translating into a Coulombic efficiency for converting CO_2_ to biomass of ca. 28%. On average, Coulombic efficiency for this process reached a level of 19.7%. With this process, a solar energy demand of 67.89 kWh kg^−1^ dry biomass could be achieved, which is competitive with photosynthesis-based processes [2]. For *K. spormannii*, a hydrogen-mediated electron transfer was postulated [13].

Despite the promise of oMES technology, several challenges limit its practical implementation [1], [19]. A critical bottleneck is the initial biofilm establishment phase, during which incomplete cathode coverage results in inefficient electron transfer and reduced productivity [2], [14]. This start-up period can extend for weeks before sufficient cathode coverage is achieved, but its duration is organism- and reactor-specific rather than a universal feature of oMES*.* At incompletely colonized cathodes, the abiotic formation of reactive oxygen species (ROS) can hamper or completely inhibit growth. This has been identified as a key limiting factor specifically for the mesophilic *C. necator,* prompting the development of strains displaying surface-exposed superoxide dismutases [17], [20]. ROS seem to be equally limiting for the thermoacidophilic *K. spormannii,* as laboratory evolution experiments provided evidence for the upregulation of mechanisms to cope with reactive oxygen species as a response to cathodic growth. Overall the sum of mutations that was acquired within this adaptive laboratory evolution approach enhanced the cathodic biofilm growth capabilities of *K. spormannii*, achieving up to fourfold higher biofilm accumulation rates in adapted populations [21]. However, conventional approaches to accelerate biofilm formation through targeted genetic engineering are not yet feasible for *K. spormannii* due to the lack of established genetic tools for this organism [1], [21].

An alternative, genetic-engineering-independent strategy for enhancing biofilm formation involves synthetic polymers that promote bacterial cell aggregation and surface attachment. Early work demonstrated that cationic polymers and dendrimers can cluster bacterial cells, thereby modulating quorum-sensing-controlled phenotypes such as bioluminescence [22], [23], [24], [25]. This concept was later extended to a pathogenic organism, where cationic polymer-induced aggregation of *Vibrio cholerae* enhanced biofilm formation while simultaneously downregulating virulence gene expression [26], [27]. Building on these findings, hydrophobic polymers were shown to increase biofilm formation in a poor biofilm-forming *E. coli* strain to levels comparable to those achieved by targeted genetic engineering, establishing synthetic polymers as a viable, mutation-free alternative to strain engineering [28]. A key methodological advance underlying this approach is the in-situ screening of poly(hydrazide) scaffolds via reversible conjugation with aldehydes [29], [30], which enables rapid optimization of polymer-microorganism interactions without isolating or purifying individual candidate polymers; the same conjugation platform has also been applied to nucleic acid delivery, underscoring its versatility [31], [32], [33]. The aldehyde moiety used for conjugation influences polymer hydrophobicity, charge distribution, and interactions with cell-surface components, providing a tunable platform for biofilm engineering [28].

In this study, we investigated whether synthetic polymers could promote cell aggregation and accelerate cathode colonization by *K. spormannii* EA-1, with the goal of testing polymer-mediated aggregation and cathode-associated layer formation as a potential strategy to address the slow start-up phase that limits this organism's use in bioelectrochemical systems, given the current lack of tools to engineer this phenotype. We screened twelve aldehydes spanning aromatic, heterocyclic, and aliphatic chemical classes for their effects on surface-attached biomass formation and cellular physiology. Promising candidates were further characterized through growth kinetics, cell viability assays, and transcriptomic analysis. Finally, we evaluated the most effective polymer-aldehyde conjugates in a continuous-flow bioelectrochemical reactor to assess their effect on cathode-associated layer formation under bioelectrochemical conditions.

## 2 Materials and methods

### 2.1 Microorganism and cultivation conditions

A *K*. *spormannii* EA-1 culture, adapted for enhanced electroautotrophic growth by adaptive laboratory evolution, was used for all experiments [21]. The strain is available from the corresponding author upon reasonable request. Heterotrophic cultivations were carried out at 60 °C and pH 6 in R2A medium with a modified composition according to Reasoner and Geldreich [34]: 5 g L^−1^ yeast extract, 1 g L^−1^ peptone, 1 g L^−1^ sodium pyruvate, 0.5 g L^−1^ casein hydrolysate, 0.5 g L^−1^ soluble starch, 0.1 g L^−1^ K_2_HPO_4_, and 10 mM MOPS buffer (pH adjusted to 6.0 with 1 M H_2_SO_4_). pH 6 was chosen for heterotrophic pre-culturing to support robust heterotrophic biomass recovery, distinct from the acidic conditions required for electroautotrophic growth.

For electroautotrophic growth, cells were cultivated at 60 °C and pH 4 in ES minimal medium (0.53 g L^−1^ NH_4_Cl, 0.15 g L^−1^ NaCl, 0.04 g L^−1^ KH_2_PO_4_, 0.12 mL L^−1^ MgSO_4_ (1 M), 1 mL L^−1^ CaCl_2_ (0.1 M), 1 mL L^−1^ Wolfe's mineral elixir; pH 4.0) with CO_2_ as the sole carbon source, matching the acidic pH range under which *K. spormannii* grows optimally in its natural environment and in cathodic biofilms [13], [16]. Heterotrophically grown precultures intended for autotrophic experiments were washed twice in ES medium (10 min at 6000 × g).

### 2.2 Chemicals and gases

Unless otherwise stated, all chemicals were purchased from Roth (Karlsruhe, Germany), VWR Chemicals (Darmstadt, Germany), Sigma-Aldrich (Munich, Germany), Merck (Darmstadt, Germany), and Fisher Scientific (Schwerte, Germany). Gases and gas mixtures were obtained from Westfalen (Münster, Germany) and HOWE Sauerstoffwerk (Steinfurt, Germany).

### 2.3 Bioelectrochemical setup

Electroautotrophic biofilm cultivation was performed in a continuous-flow fed-batch setup as previously described [14]. Microbial pre-cultures were grown in R2A-medium. For inoculation, the cells were washed in ES-medium twice, before being used for inoculation of the bioelectrochemical setup to an OD_600_ of 0.1. A smooth graphite plate was used as electrode, while iridium-tantalum coated titanium was used for the counter electrode. The process was operated at 60 °C and pH 4. The pH was stable throughout the experiment, without active pH control.

A defined dissolved oxygen concentration of 2.8 mg L^−1^ was established by gassing the headspace with pure CO_2_ and O_2_ using two mass flow controllers (Bronkhorst, Netherlands), followed by pressurization to 1.5 bar overpressure with CO_2_ for 2 min with the outlet valve being closed. The gas flow rate was set to be sufficient in order to replace the headspace volume three times. The gas phase was exchanged every 24 h. Dissolved oxygen concentration was measured non-invasively, using self-adhesive sensor spots (OXSP5-ADH; Pyroscience, Germany).

ES minimal medium was recirculated at 100 mL min^−1^. Prior to each experiment, the medium was anaerobized to remove dissolved oxygen, enabling subsequent controlled oxygen supplementation. Each experiment was initiated abiotically to establish stable electrochemical conditions before inoculation (typically after one day). All bioelectrochemical cultivations were operated chronoamperometrically.

The polycarbonate window enabled non-invasive monitoring of biofilm growth on the working electrode using optical coherence tomography (OCT). A Ganymede device (GAN200, Thorlabs, Dachau, Germany) equipped with an LSM-03 lens was used for imaging [14]. Electrochemical conditions were controlled and recorded using a potentiostat (Interface 1000B, Gamry Instruments, Warminster, USA).

### 2.4 Fluorescence microscopy

Microscopic images were acquired using a Leica DM5500B microscope equipped with a Leica K5-14401820 camera (Leica Microsystems, Wetzlar, Germany). Image acquisition was controlled using Leica Application Suite X software (version 3.8.0.26413). Samples were analyzed at 100× magnification using an HCX PL Fluotar objective (Numerical Aperture 1.30, immersion oil, 1× camera adapter Leica K5). Exposure times were adjusted per imaging session (Green/SYTO9 channel: 0.52–1.00 s; Red/PI channel: 0.32–0.50 s; phase contrast: 0.02 s, constant) while camera gain was held constant (gain = 0) across all acquisitions.

### 2.5 Polymer preparation and conjugation

#### 2.5.1 *tert*-butyl 2-(1*H*-imidazole-1-carbonyl)hydrazine-1-carboxylate

**Figure:**
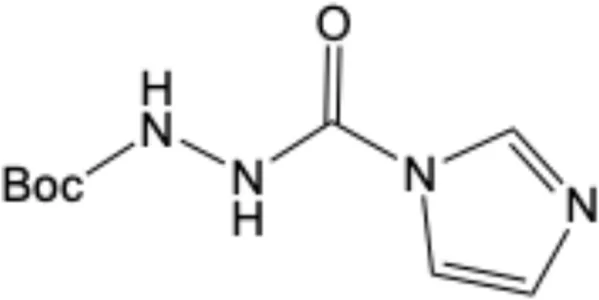

A solution of tert-butyl carbazate (595.0 mg, 4.5 mmol) was prepared in distilled water (15 mL) at room temperature, followed by cooling the mixture to 0-5 °C. Then, 1,1'-carbonyliimidazole (1459.0 mg, 9.0 mmol) was added in one portion, and the reaction mixture was stirred for 30 min until white precipitates were observed, maintaining the temperature between 0 and 5 °C. After the stirring period, the mixture was allowed to return to room temperature. The resulting solid was filtered and washed with cold distilled water (three times) and dried overnight, yielding a white powder (871.0 mg, 3.85 mmol, 85% yield).

^1^H NMR (400 MHz, DMSO-d6)/ppm: *δ* 10.42 (s, 1H, H-9, NH), 9.27 (s, 1H, H-3, NH), 8.27 (t, *J* ≈ 1.3 Hz, 1H, H-8, imidazole CH), 7.69 (t, *J* ≈ 1.5 Hz, 1H, H-5, imidazole CH), 7.09 (dd, *J* ≈ 1.7, 0.9 Hz, 1H, H-6, imidazole CH), 1.44 (s, 9H, H-12/13/14, C(CH_3_)_3_).

^13^C NMR (101 MHz, DMSO-d6)/ppm: *δ* 155.84 (C-15, OC(=O)N, Boc carbamate), 149.72 (C-2, NC(=O)N, acyl-imidazole carbonyl), 136.52 (C-8, imidazole N=CH-N), 130.48 (C-6, imidazole CH), 116.95 (C-5, imidazole CH), 80.43 (C-11, OC(CH_3_)_3_), 28.46 (C-12/13/14, OC(CH_3_)_3_).

#### 2.5.2 N-propargyl-N' - (*tert*-butoxycarbonyl)amino urea

**Figure:**
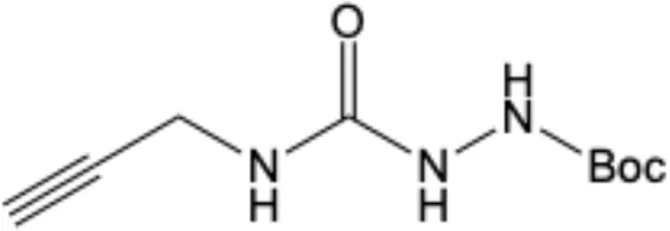

1H-imidazole-1-carbohydrazide (566.0 mg, 2.5 mmol) and propargyl amine (248.0 mg, 4.5 mmol) were each dissolved separately in ethyl acetate (15 mL) at room temperature. The propargyl amine solution was then added dropwise into the 1H-imidazole-1-1carbohydrazide, and the mixture was left to stir for 14-16 h at 30 °C. The reaction completion was monitored by thin-layer chromatography (TLC). Upon completion, the reaction was quenched with 0.5 M hydrochloric acid (15 mL). The organic layer was separated and further washed with 0.5 M hydrochloric acid (three times, 15 mL each), followed by distilled water (six times, 15 mL each), and finally a brine solution (20 mL). At each step, TLC was performed to assess appropriate isolation of the target molecule. The washed organic layer was dried over sodium sulphate and filtered. The solvent was removed using rotary evaporation to yield an off-white solid powder (429.0 mg, 2.04 mmol, 80% yield). ^1^H NMR (400 MHz, DMSO-d6)/ppm: *δ* 8.51 (s, 1H, NH), 7.75 (s, 1H, NH), 6.64 (s, 1H, NH), 3.78 (dd, *J* = 5.8, 2.5 Hz, 2H, H_2_-C≡, H-3), 3.02 (t, *J* = 2.5 Hz, 1H, ≡C-H, H-1), 1.39 (s, 9H, C(CH_3_)_3_, H-11/12/13).

^13^C NMR (101 MHz, DMSO-d6)/ppm: *δ* 158.34 (C-5, HNC(=O)NH, hydrazide carbonyl), 156.38 (C-14, OC(=O)N, Boc carbamate carbonyl), 82.79 (C-10, OC(CH_3_)_3_), 79.45 (C-2, HC ≡ C–), 72.86 (C-1, HC ≡ C–), 29.18 (C-3, HNCH_2_C≡), 28.56 (C-11/12/13, OC(CH_3_)_3_).

#### 2.5.3 poly(N-propargyl-N' -(tert-butoxycarbonyl)amino urea) pAcU-Boc

**Figure:**
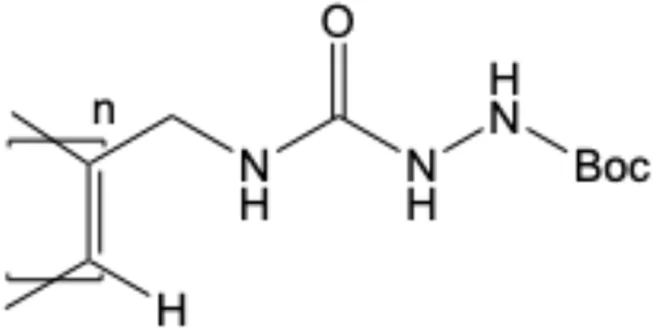

N-propargyl-N' - (tert-butoxycarbonyl)amino urea (305.0 mg, 1.43 mmol) was dissolved in chloroform (6 mL) at room temperature and degassed with argon (Ar). The mixture was heated to 30 °C until complete dissolution of the monomer**.** Thereafter, a solution of the catalyst [Rh(nbd)B(Ph)_4_] (7.0 mg, 0.0143 mmol, 0.01 equiv.) in chloroform was added to the reaction mixture dropwise with a syringe.

The reaction mixture was stirred at 30 °C for 24 h, resulting in 83% conversion. Conversion was monitored by analysing a small amount of the reaction mixture with ^1^H NMR spectroscopy at regular intervals. The product was then precipitated by adding the crude dropwise to an excess of n-hexane, and the solid form was collected after filtration. Excess solvent was removed by rotary evaporation to yield a pale-yellow powder (249.0 mg, 98% yield).

^1^H NMR (400 MHz, DMSO-d6)/ppm: *δ* 8.45 (s, 1H, NH), 7.66 (s, 1H, NH), 6.55 (s, 1H, NH), 6.10 (s, 1H, =CH), 3.76 (s, 2H, H_2_C–NH), 1.38 (s, 9H, C(CH_3_)_3_). FTIR vmax/cm^-1^; 3291 (m, H-N), 2982 (w, sh, H-C), 1710 (s, sh, C=O).

#### 2.5.4 poly(N-propargyl-N′-amino urea) pAcU

**Figure:**
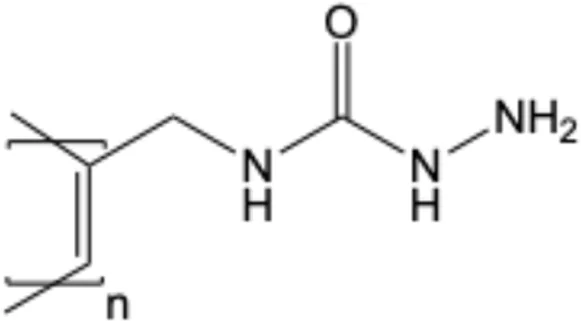

The pAcU-Boc (249 mg, 1.2 mmol) was dissolved in neat TFA (30 mg mL^−1^), and the mixture was stirred for 20-24 h at room temperature. Deprotection was confirmed by ^1^H NMR spectrum of the crude mixture. Thereafter, TFA was removed under a steady stream of argon (Ar) until obtaining a highly viscous solution, which was then carefully neutralised with sodium bicarbonate. The neutralised mixture was dialysed (Spectra/Por 6 1 kDa MWCO; 38 mm width) against deionised water in a large 5 L beaker for 4-5 days (water changed three times daily). Finally, the dialysed suspension was lyophilised to afford a pale-yellow powder, **pAcU** (236 mg, 95% yield). ^1^H NMR (400 MHz, Deuterium Oxide)/ppm: *δ* 6.08 (d, J ≈ 1.4 Hz, 1H, =CH, H-11a), 5.85 (d, J ≈ 1.4 Hz, 1H, =CH, H-11b), 3.85 (s, 2H, O-CH_2_, H-3). (NH/NH_2_ protons not observed due to exchange with D_2_O.). GPC; Mw = 41.07 g mol^-1^; Mn = 24,1 g mol^-1^; ÐGPC = 1.70.

#### 2.5.5 Post-polymerisation functionalization procedures

Separate stock solutions of pAcU (88.4 mM in monomer) in deuterated 0.2 M acetic acid and 88.4 mM aldehyde stock solutions in DMSO-d6 were prepared separately. The aldehyde stock solutions were added in equal volume to the polymer stock solutions (1 mol equiv.) and reacted at 30 °C for 24 h. The final polymer (expressed in concentration of monomer units) and aldehyde concentration was 44.2 mM. The final concentration of acetic acid was 100 mM while the final concentration fo DMSO was 50% by volume.

#### 2.5.6 Final application

Poly(hydrazide) (pAcU) stock solution was prepared by dissolving the polymer at 10 mg mL^−1^ (88.4 mmol L^−1^ in monomer) in 200 mM acetic acid with overnight incubation at 180 rpm and room temperature. Aldehyde stock solutions (88.4 mM) were prepared in dimethyl sulfoxide (DMSO). Twelve aldehydes were tested (Table 1), representing aromatic (A, B, E, F, J), heterocyclic (C, D, I), and aliphatic (G, H, K, L) chemical classes.

**Table 1:** Aldehydes used for conjugation to poly(hydrazide) (pAcU). Each aldehyde was dissolved at a concentration of 88.4 mmol L^−1^ in DMSO. Aldehydes are classified by their chemical structure into aromatic, heterocyclic, and aliphatic groups.

Reference	Aldehyde	Chemical Class
A	Benzaldehyde	Aromatic
B	2-Naphthaldehyde	Aromatic
C	1H-Imidazole-4-carbaldehyde	Heterocyclic
D	Indole-3-carboxaldehyde	Heterocyclic
E	2,3-Dihydroxybenzaldehyde	Substituted aromatic
F	5-Bromosalicylaldehyde	Substituted aromatic
G	Isovaleraldehyde	Aliphatic
H	2-Ethylhexanal	Aliphatic
I	Uracil-5-carbaldehyde	Heterocyclic
J	4-Fluorobenzaldehyde	Substituted aromatic
K	Hexanal	Aliphatic
L	Octanal	Aliphatic

For conjugation, a pAcU stock solution was mixed 1:1 with the respective aldehyde stock solution (designated PA through PL). Control preparations included: aldehydes in 200 mM acetic acid without polymer (CA through CL), untreated pAcU dissolved in DMSO (CP), and solvent control containing acetic acid and DMSO only (CS). All preparations were incubated overnight at room temperature with shaking at 180 rpm.

### 2.6 Initial screening experiment

General compatibility and enhancement of aggregated biomass formation were assessed in microtiter plates. Three microliters of each test substance (PA–PL, CA–CL, CS, CP) were added to 300 μL of culture at OD_600_ 0.05 in R2A medium, resulting in a total polymer-aldehyde-conjugate concentration of 0.44 mM in each well (expressed in concentration of monomer unit). The concentration of acetic acid per well was 0.1 mM while the concentration of DMSO-d6 was 0.5% by volume. An untreated control (CZ) was included. All conditions were tested in independent triplicates (n = 3). Growth curves were recorded at 60 °C for 92 h by measuring OD_600_ using an Infinite200Pro plate reader (Tecan Trading AG, Switzerland). The specific growth rate (μ) was calculated using Equation (1):

(1) $$\mu =\left(1/\Delta t\right)\times \ln\left({\text{OD}}_{600,t2}/{\text{OD}}_{600,t1}\right)$$

A 7-24 h interval was selected as it captured the exponential growth phase of untreated control cultures and was applied uniformly across all conditions to enable direct comparison; it does not necessarily represent the maximum growth rate (μ_max_) for conditions in which growth was delayed or extended, as observed for several polymer-aldehyde conjugate treatments.

Following growth experiments, aggregated biomass formation was quantified using crystal violet staining. Microtiter plates were carefully inverted to remove planktonic cells, and wells were washed three times with 0.1 M KH_2_PO_4_ solution, minimizing shear forces on adherent biomass. Cell aggregates were stained for 1 h at room temperature with 300 μL of 0.1% crystal violet solution (0.5 g crystal violet in 25 mL ethanol, diluted to 500 mL with double-distilled water). After removing the staining solution, wells were washed at least three times with double-distilled water and dried at room temperature. Stained aggregates were resolubilized in 300 μL of 33% acetic acid and absorbance was measured at 550 nm. Of note, crystal violet is not cell-specific and can be retained by polymer deposits; the assay was therefore used as an exploratory, relative measure.

### 2.7 Live/dead staining

Based on screening results, four aldehydes (B, C, I, K) were selected for viability assessment. Cells were incubated with polymer-aldehyde conjugates for four days as described above. Live/dead staining was performed by adding 1 μL SYTO9 (5 mM stock; ThermoFisher Scientific) and 1.5 μL propidium iodide (1 mg mL^−1^ stock; ThermoFisher Scientific) per 1 mL cell culture, followed by 20 min incubation in darkness. Cells were washed three times with ES medium (16,000 × g, 1 min) to remove excess unbound stain and reduce background fluorescence prior to imaging. This centrifugation protocol was applied uniformly across all conditions, ensuring any potential loss or disruption of aggregates would affect all samples comparably. Images were acquired using L5 (SYTO9) and Y3 (propidium iodide) fluorescence filters [35]. Polymer-aldehyde conjugate aggregates exhibited autofluorescence in the same emission channels used for SYTO9 and propidium iodide detection; as no spectral unmixing or software-based signal separation was performed, cell counting for live/dead ratio determination was instead performed manually, distinguishing cells from polymer autofluorescence based on morphological criteria (cell size and shape) under visual inspection of three independent microscopy images. Given the informal nature of this discrimination, live/dead ratios are presented as an approximate, relative assessment of cellular response rather than a precisely validated quantitative measure.

### 2.8 Transcriptomic analysis

Fresh cultures grown in R2A medium were adjusted to OD_600_ 1.0. In duplicates, 100 μL of polymer-aldehyde conjugates (B, C, or K) was added to 500 μL of culture. Control cultures received no polymer addition. After 4 h incubation at 60 °C, 600 μL RNAprotect (Qiagen, Netherlands) was added to stabilize RNA. RNA extraction was performed using the RNeasy PowerBiofilm Kit (Qiagen). Extracted RNA was stored at −80 °C. Reverse transcription, rRNA depletion, library preparation and Illumina sequencing were performed by Eurofins Genomics, Ebersberg, Germany. Directional and paired-end sequencing was performed on a NovaSeq X. Read sequences were analyzed with the Qiagen CLC Genomics Workbench v20 using the RNA-Seq analysis workflow and standard parameters (Mismatch cost 2, insertion cost 3, deletion cost 3, length fraction 0.8, similartiy fraction 0.8, no global alignment) with the *Kyrpida spormannii* reference genome (CP024955). Expression values were calculated as TPM, Venn Diagram is based on FDR <0.05-filtered values.

Heterotrophic conditions (R2A medium, pH 6) were used for the initial screening and for transcriptomic profiling, as the substantially faster growth rate under heterotrophic conditions compared to electroautotrophic CO_2_-fixing growth allowed practical throughput for testing multiple polymer-aldehyde conjugates and biological replicates within a feasible timeframe. The lead candidate identified through this screening approach (conjugate B) was subsequently evaluated under electroautotrophic conditions in the bioelectrochemical reactor, confirming that the polymer-mediated effect on cathode-associated layer formation is also observed under the intended process conditions.

### 2.9 Bioelectrochemical system experiment

The effect of polymer addition on aggregate formation in the bioelectrochemical system was evaluated using three parallel setups: (1) biotic with polymer-aldehyde conjugate, (2) biotic control without polymer, and (3) abiotic control with polymer-aldehyde conjugate but no cells. All reactors were operated at −500 mV vs. SHE. For these experiments, the final polymer-aldehyde conjugate concentration was reduced from 0.44 mM to 0.088 mM (expressed in concentration of monomer units). Biomass aggregates or cathode-layer height was measured daily using OCT at one fixed position on each electrode (middle), consistent with the central position defined by Hackbarth et al. [14]. By cutting these images into three equal pieces of 2 × 4 mm, mean values and standard deviations could be calculated. Biotic setups were initially operated abiotically before inoculation to OD_600_ 0.1 after one day; inoculum cultures were prepared as described in the Microorganism section. Polymer-aldehyde conjugate was added simultaneously with cells. Each of the three conditions was tested in a single bioelectrochemical reactor (n = 1).

### 2.10 Statistical analysis

Statistical analysis was performed in Excel (Microsoft, Redmond, USA). The crystal violet assay and the planktonic growth-rate determination were performed with n = 3 independent biological cultures per condition. Data are presented as mean ± standard deviation. For each aldehyde, the polymer-aldehyde conjugate treatment was compared against the respective aldehyde-only control, using a two-tailed, unequal-variance (Welch's) *t*-test (Excel T.TEST function). To account for testing four candidates, a Bonferroni-corrected significance threshold of α = 0.0125 (0.05/4) was applied.

For transcriptomic principal component analysis (n = 2 per condition), within-group dispersion was quantified as the Euclidean distance between replicate pairs in PC1–PC2 space rather than by formal hypothesis testing, given the limited replicate number.

## 3 Results

For *E*. *coli* it was shown that the addition of a polymer-aldehyde conjugate had a positive effect on biofilm formation [28]. The aim of this study was to investigate whether the addition of such polymer to *K. spormannii* cultures would have a similar effect and accelerate growth on cathode surfaces. Candidate polymers were prepared by conjugating a reactive polymer (pAcU) with an aldehyde out of a list of twelve to give it a specific property (Table 1). Based on previous work, we targeted hydrophobic aldehydes including aliphatic aldehydes (G, H, K, L), aromatic aldehydes (A, B, E, F, J), and heterocyclic aldehydes (C, D, I). We anticipated that all these aldehydes could have different interactions with the cells.

### 3.1 Aldehyde screening for enhanced aggregate formation

To investigate the effect of polymer-aldehyde conjugates on aggregate formation, cell cultures were incubated for four days under controlled conditions. The experimental design included the following treatment groups: (1) polymer-aldehyde conjugates, (2) aldehyde only (negative control), (3) untreated polymer (pAcU), (4) solvent controls containing acetic acid and dimethyl sulfoxide (DMSO), and (5) untreated controls.

**Fig. 1:**
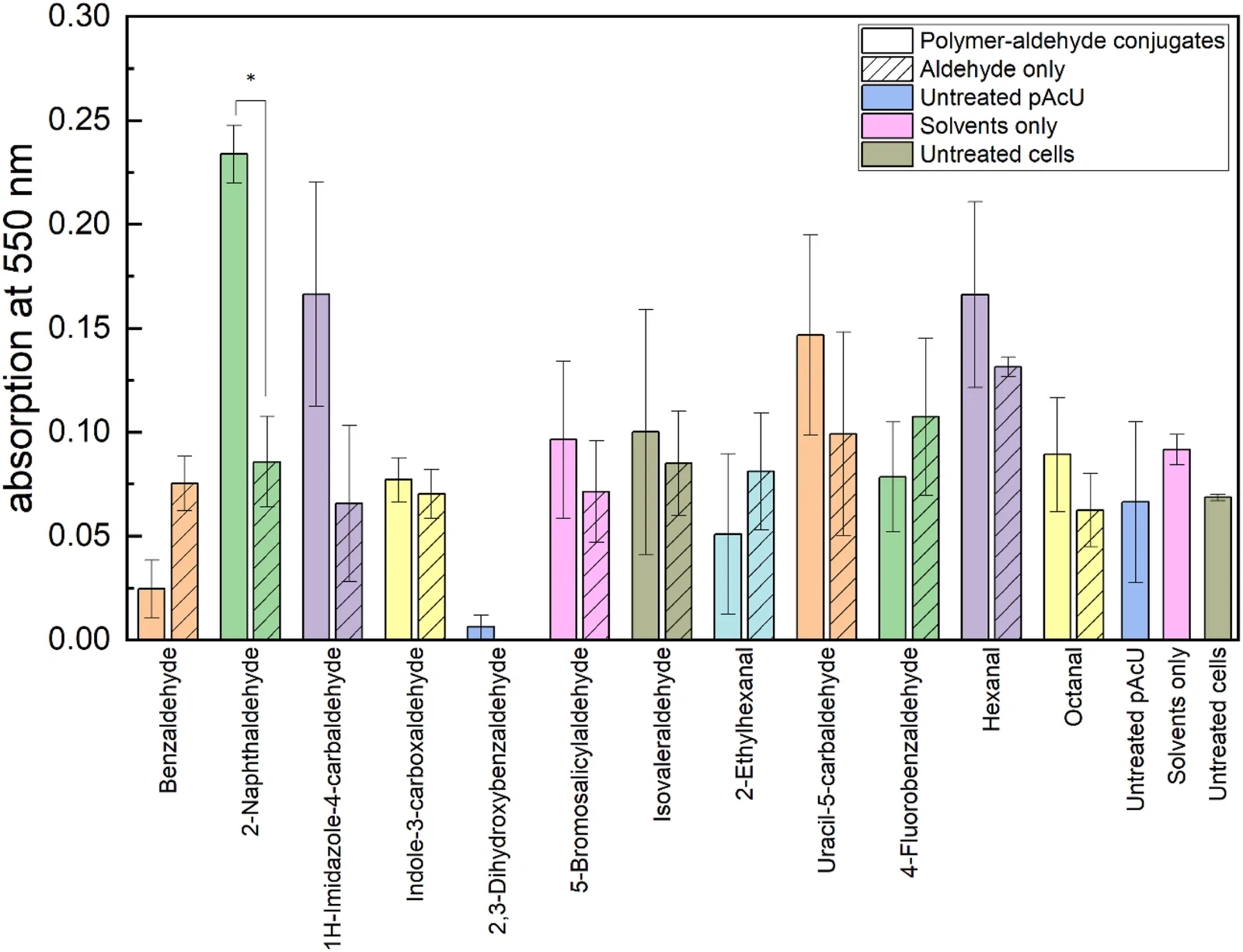
Assessment of aggregate formation using crystal violet staining. Cell cultures were incubated with polymer-aldehyde conjugates (unshaded columns, A–L) or controls: aldehyde only (hatched columns, A–L), untreated pAcU (CP), solvents only (CS), or untreated cells (CZ). After 92 h, aggregates were stained with crystal violet and absorbance was measured at 550 nm. Data represent mean ± standard deviation of n = 3 independent biological cultures. (For interpretation of the references to color in this figure legend, the reader is referred to the Web version of this article.)

Following a 92-h incubation period, planktonic cells were removed by gentle washing, and adherent aggregates formed on microtiter plate wells were stained using the crystal violet assay (Fig. 1).

All conjugates showing visibly higher absorbance than their respective aldehyde-only controls were carried forward for further characterization. This was particularly true for aldehydes B (2-naphthaldehyde, aromatic), C (1H-imidazole-4-carbaldehyde), I (uracil-5-carbaldehyde), both heterocyclic, and K (hexanal, aliphatic). Subsequent statistical comparison of each selected conjugate against its aldehyde-only control (two-tailed *t*-test, Bonferroni-corrected threshold α = 0.0125) confirmed a significant difference for B (p = 0.0025), while C, I and K did not reach statistical significance relative to aldehyde-only (p = 0.104; p = 0.384 and p = 0.387, respectively), indicating that for these three candidates the aldehyde component alone likely accounts for a substantial part of the observed effect. Nevertheless, B, C, I, and K were retained for further characterization based on their consistent visual increase in absorbance across replicates, warranting investigation of their effects on growth kinetics, cell viability, and gene expression. For 2-naphthaldehyde (B), absorbance was highest at 0.23 ± 0.01.

### 3.2 Growth kinetics of selected aldehydes

The impact of the polymer-aldehyde conjugates on cellular fitness was assessed by quantifying planktonic growth. B (2-naphthaldehyde), C (1H-imidazole-4-carbaldehyde), I (uracil-5-carbaldehyde) and K (hexanal) were selected based on the exploratory crystal-violet screen. Optical density at 600 nm (OD_600_) was monitored over 95 h to generate growth curves (Fig. S1). Control groups included cultures treated with aldehyde alone, untreated polymer (pAcU), solvent controls (acetic acid and DMSO), and untreated controls.

**Fig. 2:**
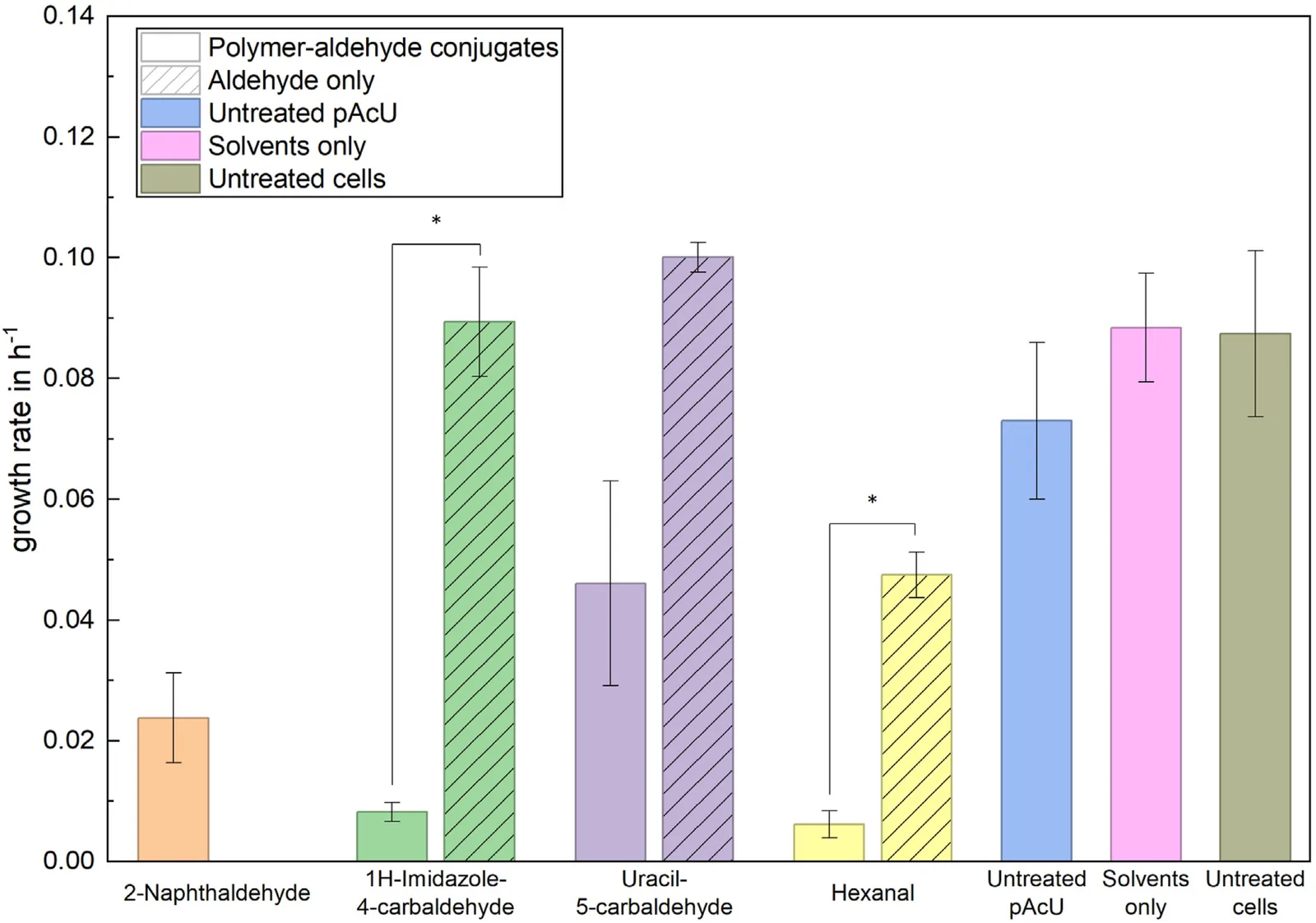
Specific apparent growth rates of planktonic cell cultures after addition of polymer-aldehyde conjugate. Growth rates were calculated between 7 h and 24 h after inoculation. Controls include the addition of the respective aldehyde only (shaded columns), polymer only (CP), solvents only (CS), and untreated cells (CZ). Growth curves are provided in Supplementary Fig. S1. Data represent mean ± standard deviation of n = 3 independent biological cultures.

Growth curves comprising ten sampling points over 95 h were recorded for all conditions and controls (Fig. S1). As OD_600_ is affected by aggregation, sedimentation, and light scattering from the polymer-aldehyde preparations, the OD-based growth rates reported here are apparent values that reflect changes in optical density rather than validated, direct measurements of cell division (Fig. 2). Following an initial phase of low optical-density increase (∼7 h), untreated cell cultures showed the highest apparent growth rate (μ = 0.09 h^−1^), comparable to solvent control cultures. In contrast, cultures containing polymer-aldehyde conjugates demonstrated reduced apparent growth rates during the first 24 h: 0.02 h^−1^ (aldehyde B), 0.01 h^−1^ (aldehyde C), 0.04 h^−1^ (aldehyde I), and 0.01 h^−1^ (aldehyde K). Statistical comparison against the respective aldehyde-only controls (two-tailed *t*-test, Bonferroni-corrected threshold α = 0.0125) confirmed significantly reduced apparent growth rates for C (p = 0.0032) and K (p = 0.0002), while the difference for I did not reach statistical significance (p = 0.041). For B, the apparent growth rate of aldehyde-only control could not be determined during the relevant interval, precluding a formal statistical comparison. The conjugate-treated culture's consistently positive apparent growth rate across replicates is nonetheless reported as a clear qualitative difference. Despite this reduced early growth, cultures containing polymer-aldehyde conjugates showed a continued, slower increase in optical density over the remainder of the cultivation; a clearly defined stationary phase was not reached within 95 h (Fig. S1). As growth rates were calculated over a fixed early interval (7–24 h) rather than per-condition maxima, the reported values for polymer-aldehyde conjugate treatments likely underestimate the true maximum growth rate reached later in cultivation for these delayed-growth conditions.

### 3.3 Live/dead staining

**Fig. 3:**
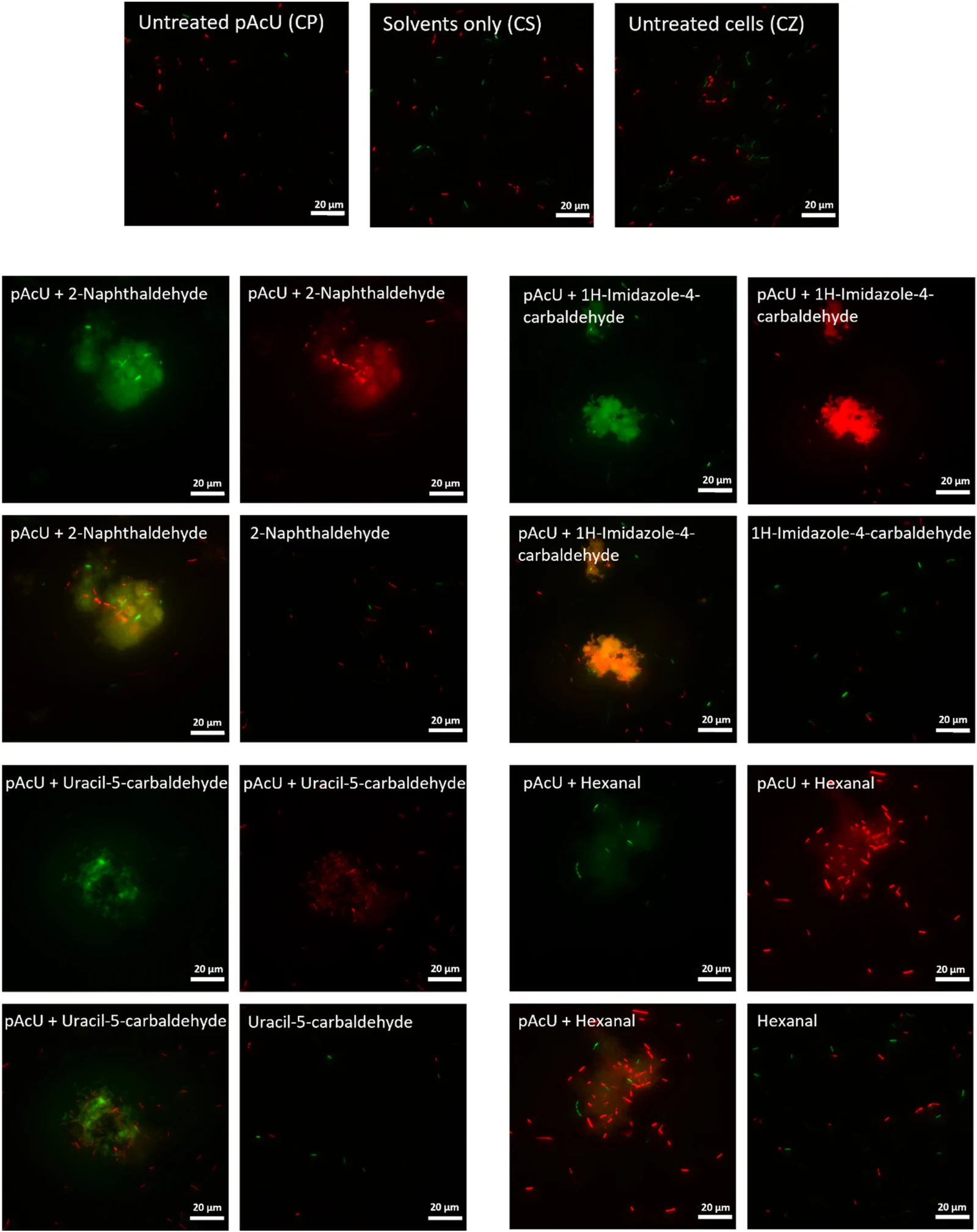
Live/dead fluorescence staining after aggregate formation in the presence of polymer-aldehyde conjugates. The polymer was functionalized with B (2-naphthaldehyde), C (1H-imidazole-4-carbaldehyde), I (uracil-5-carbaldehyde) and K (hexanal). Cells stained green with SYTO9 (upper left panels) are viable, while cells stained red with propidium iodide (upper right panels) are non-viable. Lower left panels show merged channels. Lower right panels show aldehyde-only controls. Additional controls (untreated polymer (pAcU), solvents only, untreated) are shown in the top row. Images were acquired at 100× magnification. The images were chosen as a representative example of at least three independent images. (For interpretation of the references to color in this figure legend, the reader is referred to the Web version of this article.)

Cell viability was assessed following growth experiments using live/dead fluorescent staining. Fig. 3 shows the microscopic images for the aldehydes B (2-naphthaldehyde), C (1H-imidazole-4-carbaldehyde), I (uracil-5-carbaldehyde) and K (hexanal), as well as all negative controls.

**Fig. 4:**
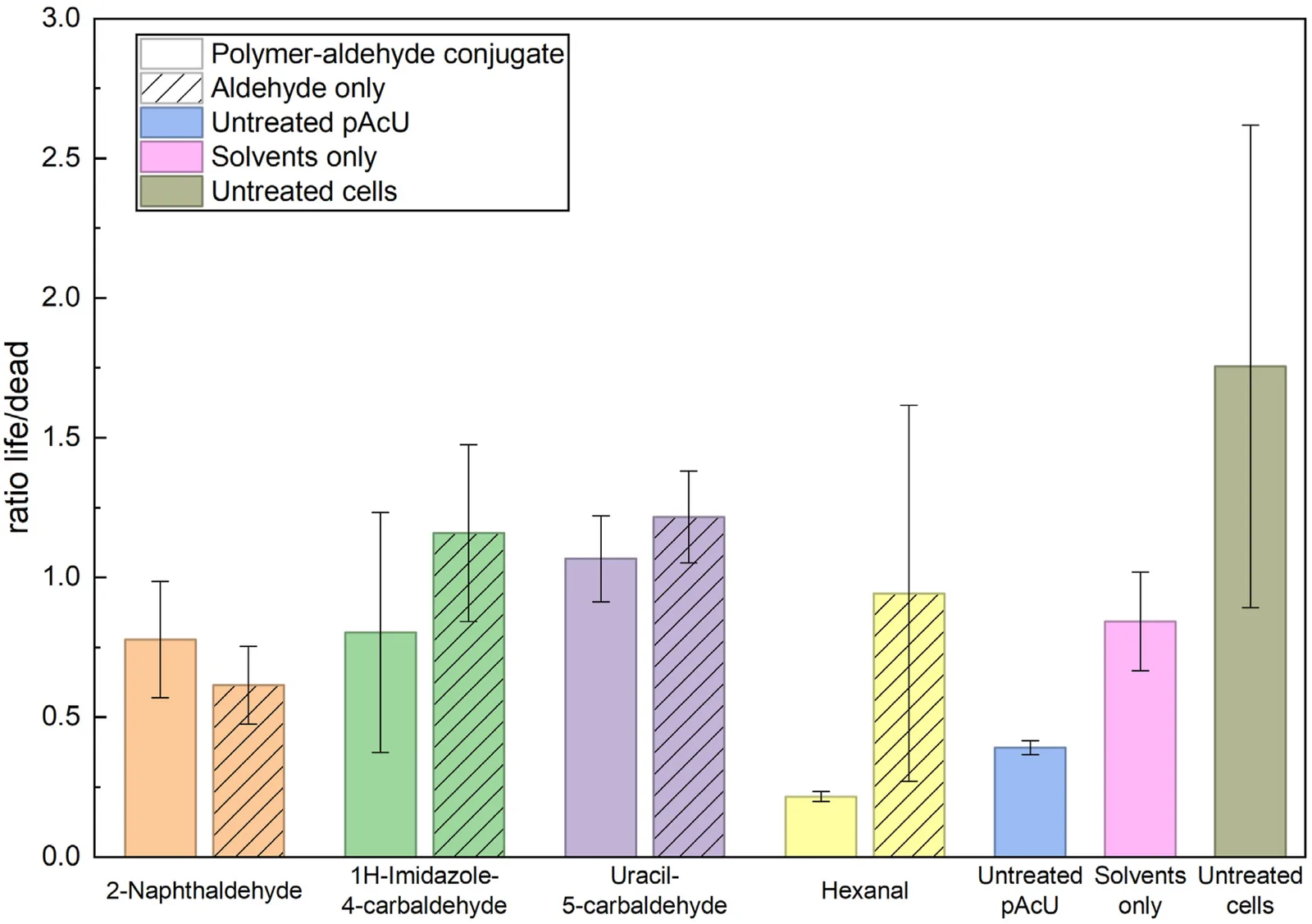
Quantification of live/dead cell ratios after growth with polymer addition. The ratio of viable to non-viable cells was determined by manual cell counting from fluorescence microscopy images, distinguishing cells from polymer autofluorescence based on morphology. Controls include the addition of the respective aldehyde only (shaded columns), untreated polymer (pAcU) (CP), solvents only (CS), and untreated cells (CZ). Data represent mean ± standard deviation of three independent microscopy images (n = 3).

Fluorescence microscopy revealed distinct cellular responses depending on the aldehyde used for conjugation. Polymer-aldehyde conjugates formed distinctive clusters that were absent in all control conditions. These clusters exhibited autofluorescence at the experimental wavelengths, resulting in visible coloration in microscopic images. Cells were distinguished from polymer autofluorescence manually, based on characteristic cell morphology. Based on this assessment, cell-cluster interactions appeared to vary among aldehyde types: 2-naphthaldehyde (B) and uracil-5-carbaldehyde (I) appeared to promote cellular attachment with mixed viability (both viable and non-viable cells present), hexanal (K) appeared to show predominantly non-viable attached cells, while 1H-imidazole-4-carbaldehyde (C) showed no detectable cellular attachment to polymer conjugates. The ratio of live cells to dead cells was determined by cell counting (Fig. 4).

For all aldehydes except aldehyde B, the ratio of live cells to dead cells was higher in the controls without polymer addition. Even though the difference was not significant, there appears to be a trend towards more harmful effects of the polymer-aldehyde conjugate compared to aldehyde only. The ratio of live to dead cells was highest for the control culture without any additions.

### 3.4 Transcriptomic response to polymer-aldehyde conjugate treatment

**Fig. 5:**
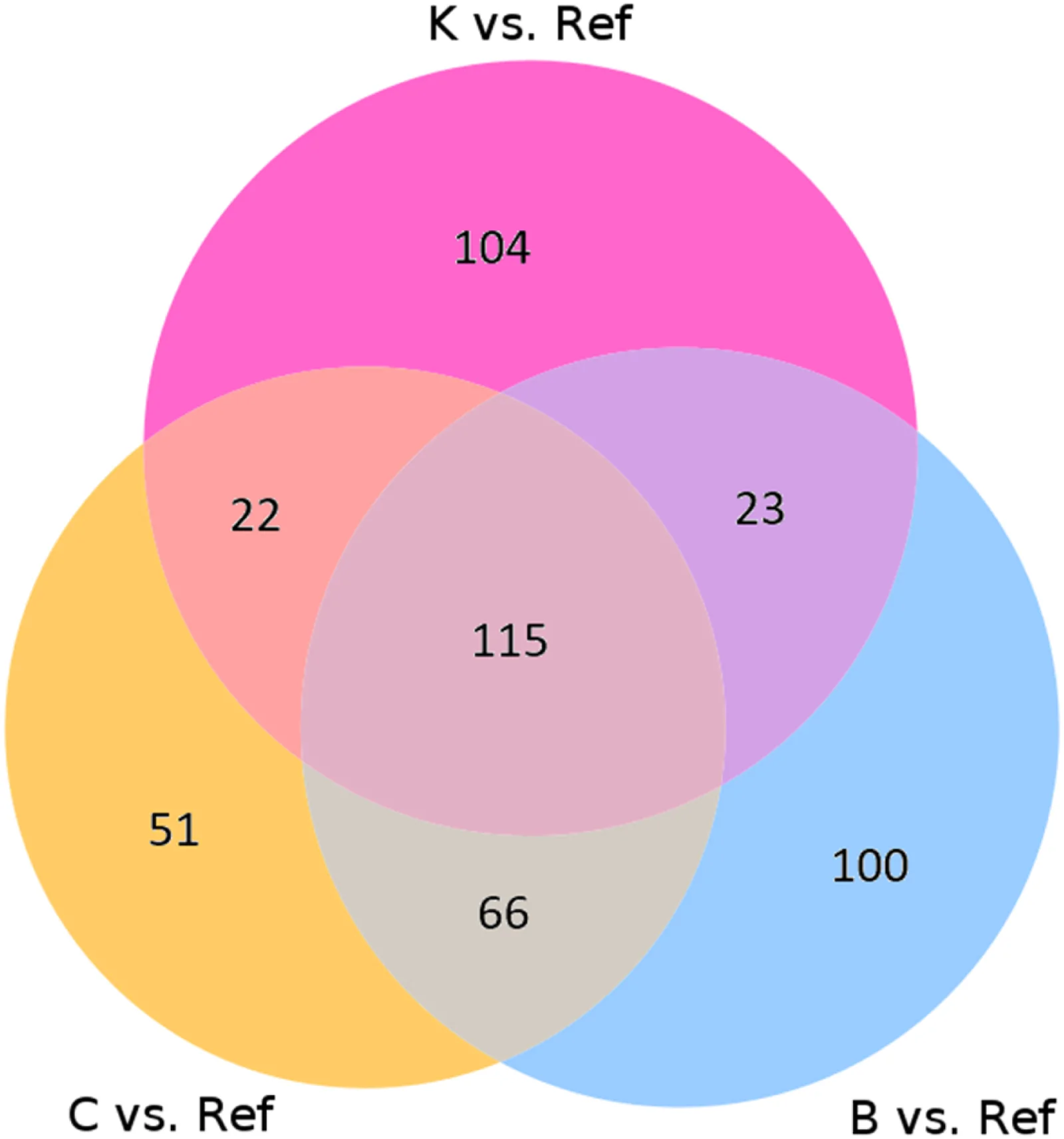
Venn diagram of differentially expressed genes from transcriptome analysis. All genes that were up- or down-regulated by at least a factor of 5 compared to the untreated reference are included. Numbers indicate genes uniquely or commonly regulated across treatments with polymer aldehyde B-, C-, or K-conjugates. The data were not fdr-filtered.

As treatment with 2-naphthaldehyde (B), 1H-imidazole-4-carbaldehyde (C) and hexanal (K) led to a pronounced aggregation phenotype we examined whether a common transcriptional response accompanied conjugate treatment. Principal component analysis separated the four experimental conditions along the first two principal components, together accounting for 69.9% of the total variance (Fig. S2). Correlation coefficients within each replicate are 0.95 (B) 0.96 (C) 0.96 (K) and 0.98 (reference). Applying a fold-change threshold of ≥5 and FDR filter <0.05, 46 genes were consistently upregulated and 51 consistently downregulated across all three aldehyde treatments, with additional condition-specific responses (Fig. 5, Supplementary Table S1).

The downregulation of flagellar motility was the most prominent and consistent change across all three conditions. Structural and regulatory components spanning the synthesis (CVV65_08815 - CVV65_08825, CVV65_08840, CVV65_08845, CVV65_14225, CVV65_08900), export apparatus (CVV65_14215), motor switch (CVV65_08855, CVV65_08860), basal body (CVV65_08865, CVV65_08875), hook (CVV65_14715), assembly (CVV65_14700) and anti-sigma factor (CVV65_14725) were all significantly reduced. Flagellin itself (CVV65_14690) was also suppressed in two of three treatments. This concerted downregulation indicates a coordinated repression of flagellar gene expression under the conditions tested. In line with this typical response to an adherent lifestyle is the apparent change in the polysaccharides chemistry which is possibly mediated by the five polysaccharide deacetylase family genes that were consistently induced across conditions (CVV65_00970, CVV65_02700, CVV65_2705, CVV65_06560, CVV65_15260) including the sporulation-associated PdaB (CVV65_00970).

Components of the type IV pili assembly system were upregulated (CVV65_15460, CVV65_15465), while the twitching motility ATPase PilT (CVV65_04775), which drives pilus retraction, was downregulated.

The master developmental transcription factor Spo0A (CVV65_05170) and its sigma factor SigH (CVV65_00700) were induced across all treatments, alongside multiple downstream sporulation-associated membrane proteins, the stage IV sporulation protein SpoIVA (CVV65_07695), and the AbrB antagonist Veg (CVV65_00325). Several of these targets showed among the highest fold-changes in the entire dataset and were consistently upregulated across all three aldehyde conditions.

An ECF sigma factor with homology to SigW (CVV65_02285), together with its cognate anti-sigma factor RsiW (CVV65_02280), was strongly and consistently induced. A second ECF sigma factor showed particularly high induction by aldehyde C and K treatments (CVV65_05770). These genes are hallmarks of cell envelope perturbation signaling.

Three phosphodiesterase genes degrading c-di-GMP were upregulated (CVV65_05765, CVV65_14050, CVV65_14465), including an HD-GYP domain protein that was among the most consistently overexpressed genes across all strains. Conversely, a diguanylate cyclase responsible for c-di-GMP synthesis was slightly downregulated (CVV65_02455).

A type II RelE/ParE toxin-antitoxin system (CVV65_15000) was strongly induced, most prominently by aldehyde B treatment. This might be the reason for the extreme fold-changes in the dataset observed for catabolic gene clusters encoding branched-chain amino acid transporters (CVV65_14950 – CVV65_14960, CVV65_08250, CVV65_08540) and degradation enzymes (CVV65_12340 – CVV65_12350), pyruvate dehydrogenase (CVV65_10295), alpha-ketoacid dehydrogenase complexes (CVV65_10290), carbon monoxide dehydrogenase subunits (CVV65_13175, CVV65_13185, CVV65_13920, CVV65_13925), and ethanolamine utilization genes (CVV65_12970, CVV65_12990, CVV65_13000, CVV65_13005), all of which were consistently and massively downregulated across treatments (Supplementary Table S1).

The number of all genes that were up- or down-regulated by at least a factor of five relative to the reference is shown in a Venn diagram (Fig. 5). The genes concerned are listed in Supplementary Table S1.

### 3.5 Cathode-associated layer formation in the bioelectrochemical system

In a final experiment, the effect of adding a polymer-aldehyde conjugate to cultivation in an oMES process was investigated. 2-naphthaldehyde (B) was selected on the basis of the combined screening criteria: it was the only conjugate with a statistically significant increase in crystal-violet-retained biomass over its aldehyde-only control (p = 0.0025), and, unlike hexanal (K), it maintained cell viability in the live/dead analysis. Three parallel approaches were started: (1) biotic with polymer functionalized with 2-naphthaldehyde added together with cells, (2) biotic negative control with cells but no polymer, and (3) abiotic negative control with polymer-aldehyde conjugate but no cells. The cultivation conditions remained unchanged between the three approaches.

**Fig. 6:**
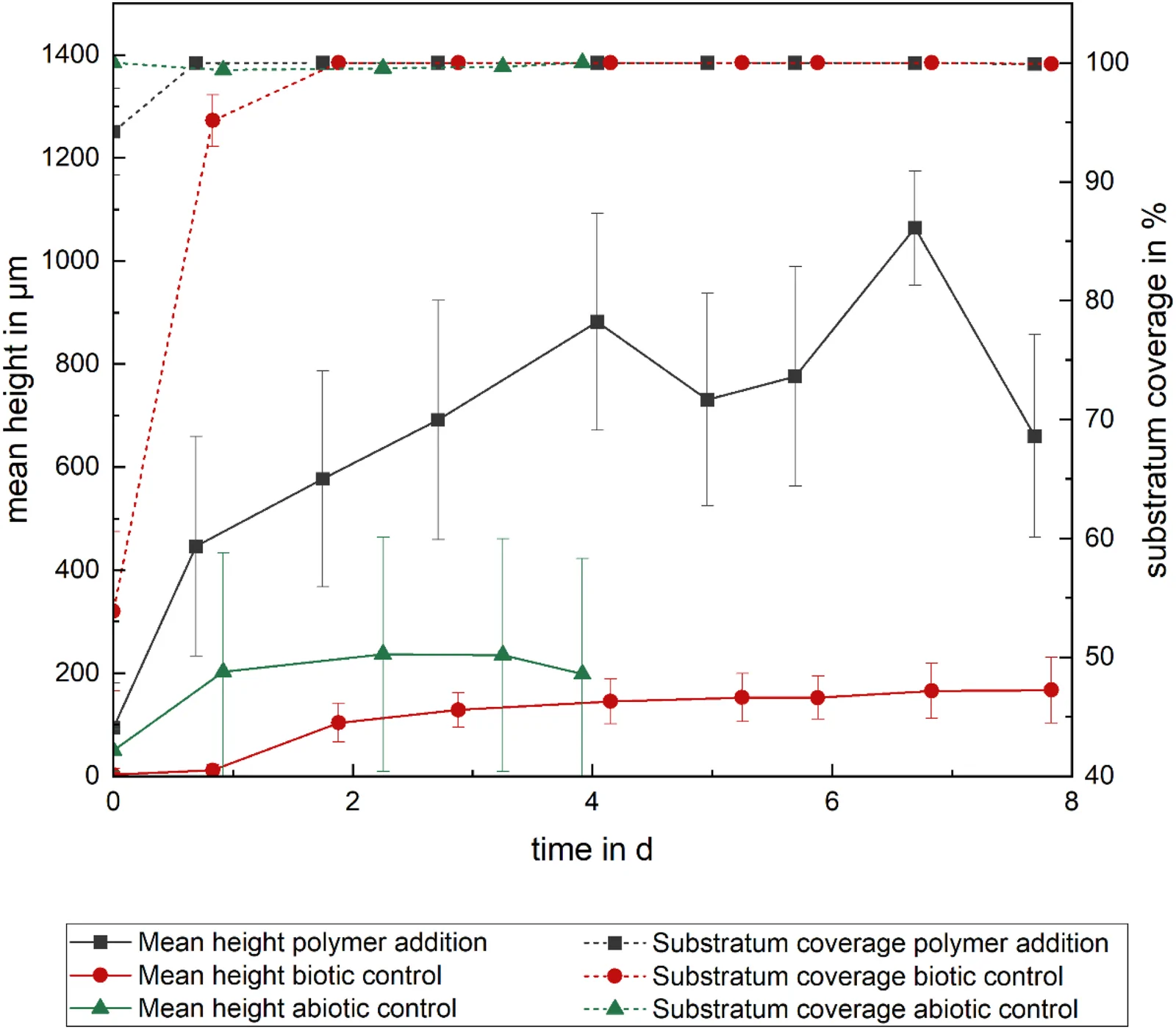
Cathode-associated layer development in the bioelectrochemical system with polymer addition. Mean layer height (solid lines) and substratum coverage (dashed lines) are shown for: biotic culture with polymer-2-naphthaldehyde (B) conjugate (gray), biotic control without polymer (red), and abiotic control with polymer-2-naphthaldehyde (B) conjugate but no cells (green). Imaging parameters were derived from daily OCT imaging. The experiments were performed in single reactors (n = 1). Data represent mean ± standard deviation of three OCT images, right next to each other in the middle of the electrode. (For interpretation of the references to color in this figure legend, the reader is referred to the Web version of this article.)

Daily OCT images quickly revealed that the addition of the polymer-2-naphthaldehyde (B) conjugate, both biotically and abiotically, caused a strong accumulation of large aggregates on the cathode. The layer was so thick that the light beam emitted by the OCT device could no longer fully penetrate it from day 2, which meant that the volume was under-quantified. However, the surface of the biomass aggregates or polymer structures was not affected by this limitation and could be visualized accurately in the OCT images. As a result, the average height of the cathodic layer and the degree of coverage of the cathode could be derived (Fig. 6).

The development of the mean layer height in the biotic culture incubated with the polymer-2-naphthaldehyde (B) conjugate exceeded both control conditions. The maximum was over 1 mm after about seven days, compared to 0.17 mm in the biotic control (without polymer) after almost eight days, and 0.24 mm in the abiotic, cell-free control (polymer only) after three days. As the abiotic polymer-only control reached a greater layer height than the biotic control without polymer, this indicates that polymer deposition alone, independent of cellular activity, contributes substantially to the layer height measured by OCT. The layer height reported here therefore reflects the combined contribution of cellular biomass and polymer material rather than cell-specific biofilm biomass. Notably, the combination of cells and polymer-aldehyde conjugate together produced a substantially greater layer height than either component alone, exceeding both the abiotic polymer-only control and the biotic control without polymer, indicating that the presence of cells and polymer together promotes greater layer formation than either alone. Complete electrode coverage was achieved more quickly with the addition of the polymer-aldehyde conjugate than in the biotic control without polymer addition, taking approximately two days. As this experiment was performed with a single reactor per condition (n = 1), these observations are presented as a qualitative comparison rather than a statistically validated result.

**Fig. 7:**
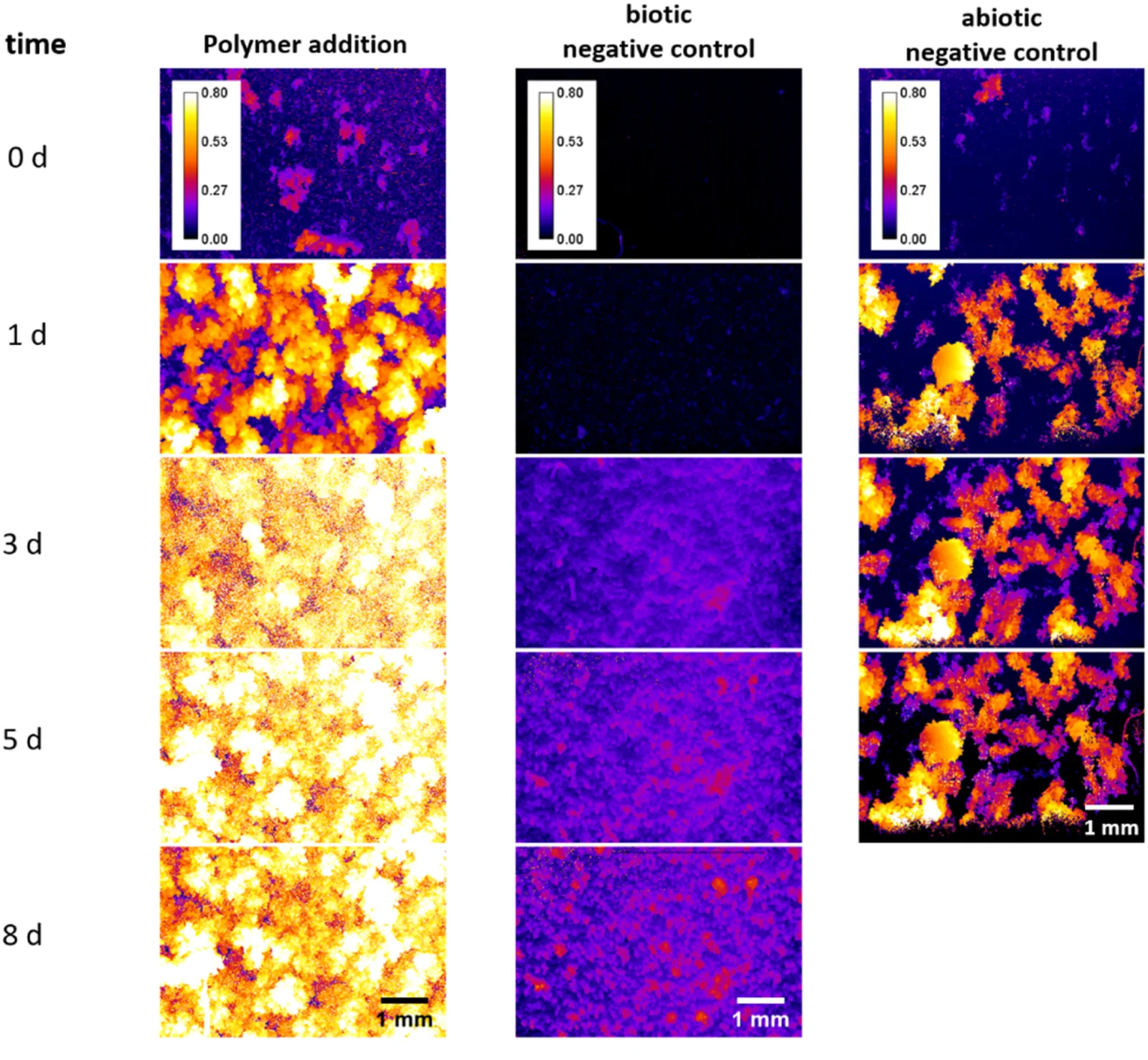
Height maps of polymer/cell layers in the bioelectrochemical system. Top views are shown for biotic culture with polymer-2-naphthaldehyde (B) conjugate (left row), biotic control without polymer (middle row), and abiotic control with polymer-2-naphthaldehyde (B) conjugate (right row) directly after inoculation and after 1, 3, 5, and 8 days of cultivation. Color scale indicates height in mm and saturates above 0.80 mm. (For interpretation of the references to color in this figure legend, the reader is referred to the Web version of this article.)

The height profiles (Fig. 7) provide information about the uppermost layer of the structures. It is noticeable that the height and structure of the layers remained approximately constant with the addition of polymer-2-naphthaldehyde (B) conjugate after just three days, as did the height of the polymer structures in the abiotic negative control, while the height of the biofilm in the biotic negative control continued to change until day eight.

**Fig. 8:**
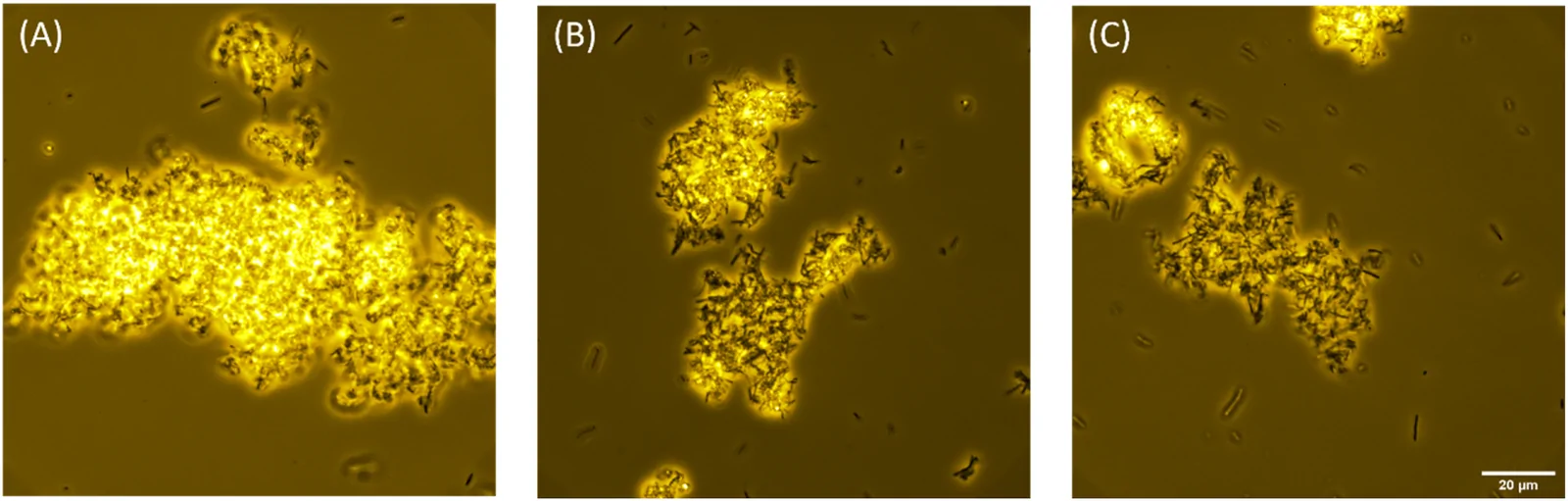
Phase contrast microscopy of polymer-cell agglomerates from the biotic culture with polymer-2-naphthaldehyde (B) conjugate in a bioelectrochemical system. Samples were taken at the (A) front, (B) center, and (C) rear measurement positions on the cathode. A color filter was applied to improve visibility of cellular structures within the polymer matrix. (For interpretation of the references to color in this figure legend, the reader is referred to the Web version of this article.)

After the experiment was completed, samples of the biomass aggregates to which the polymer-2-naphthaldehyde (B) conjugate had been added were examined under a microscope. Samples from the front end, center, and rear end of the electrode were examined using phase contrast microscopy (Fig. 8). Large agglomerates of the polymer had formed, in which many cells were enclosed. These images demonstrate cellular entrapment within the polymer matrix but do not establish whether entrapped cells were growing or metabolically active.

## 4 Discussion

The aim of this study was to develop a new strategy for improving biofilm formation in *K. spormannii* without the need for genetic modification. The approach was based on previous work by Adoni et al., that demonstrated that hydrophobic polymers could enhance biofilm formation in *E. coli* [28]. The underlying mechanism involves the aggregation of the bacteria in the presence of the polymers, and the activation of critical phenotypes as a result of this aggregation [22], [23], [25], [26], [27]. The addition of hydrophobic polymers achieved, in strain MC4100, biofilm levels comparable to strain PHL644, which carries a point mutation that allows enhanced biofilm formation, demonstrating that polymer-based approaches can substitute for genetic modification [28]. Since genetic modification of *K. spormannii* is not yet possible due to the lack of established genetic systems, this method represents an attractive alternative for accelerating biofilm formation, particularly during the energetically inefficient start-up phase characterized by incomplete electrode coverage.

In an initial study, it was first investigated which polymer-aldehyde conjugates could improve aggregate formation. From the control approaches compared to the pure cell culture (CZ), neither the solvents nor the untreated polymer nor the aldehydes alone strongly inhibited planktonic growth. This is distinct from the polymer-aldehyde conjugate preparations themselves, which reduced apparent planktonic growth rates and, for some aldehydes, cell viability (see below); the absence of an inhibitory effect of the individual components therefore does not extend to the conjugates. From the assessment of aggregate formation using crystal violet staining after incubation with the polymer-aldehyde conjugates, four candidate aldehydes were selected for further investigation: 2-naphthaldehyde (B) – aromatic, 1H-imidazole-4-carbaldehyde (C) and uracil-5-carbaldehyde (I) – heterocyclic, and hexanal (K) – aliphatic. Of these, only conjugate B produced a crystal-violet signal significantly higher than its aldehyde-only control, whereas for C, I and K the aldehyde-only controls were comparable, indicating that the aldehyde component alone likely accounts for a substantial part of the signal for these three candidates. As in the study on *E. coli*, aromatic aldehydes tended to be more effective than aliphatic ones [28].

The apparent planktonic growth rate, an OD-based measure potentially confounded by polymer-induced aggregation, sedimentation, and light scattering (see Results), was generally lower in the approaches to which polymer-aldehyde conjugates were added compared to the controls. This may indicate either a growth-inhibiting effect or that growth occurred to some extent in the form of biofilms on the walls of the wells. Live/dead staining was then used to check whether the polymer, aldehyde, or solvents could have harmful effects on the cells. The images showed that cells became enclosed within the polymer aggregates. Furthermore, for aldehyde K (hexanal) it was observed that a large proportion of the cells entrapped by the polymer were dead. Hexanal is a known antimicrobial agent that damages cell membranes and thus induces cell death [36]. As live/dead staining was performed at a single timepoint four days after polymer addition, these data reflect a late-stage snapshot and may be biased toward accumulated cell death over this period; they do not describe viability during early attachment or under actual bioelectrochemical reactor conditions.

The transcriptome data were obtained 4 h after polymer addition from whole cultures without component-resolved controls; they capture a global, acute response to conjugate exposure that is compatible with — but does not establish — a transition from a planktonic to a sessile lifestyle, and is equally compatible with acute chemical and cell-envelope stress. The interpretations below are presented as hypotheses. The coordinated repression of flagellar genes could mirror the canonical motility-to-biofilm switch documented in diverse bacteria, in which flagella are dismantled upon surface contact to redirect resources toward biofilm matrix production [37]. The simultaneous induction of prepilin components alongside downregulation of the pilus retraction ATPase PilT is, at the transcript level, compatible with a shift in the balance between pilus extension and retraction, which in other bacteria accompanies the transition from exploratory motility to surface attachment [38].

The induction of Spo0A and SigH is a key finding with direct relevance to potential biofilm formation in *K. spormannii*. In *Bacillus subtilis* and related Firmicutes, low-level Spo0A phosphorylation represses the transition regulator AbrB, activating biofilm matrix genes rather than committing cells to sporulation [39]. Still, an in-depth analysis of phosphorylation status would be necessary to clearly assign the detected upregulation to this function. The concurrent strong induction of the AbrB antagonist Veg suggests an analogous derepression of biofilm loci here [40], [41]. The broad upregulation of sporulation-associated genes could reflect the transcriptional pleiotropism of moderate Spo0A∼P levels, which activate developmental gene sets without triggering terminal sporulation [39].

The ECF sigma factor response is consistent with the polymer-aldehyde conjugates causing cell envelope perturbation upon contact [42]. In *B. subtilis* membrane disturbance activates SigW/RsiW signaling, which drives cell wall modification and downstream adaptation responses [43], [44]. This pathway may therefore act as an early sensor of polymer contact. Whether it is coupled to attachment-related gene expression, or instead reflects a generic envelope-stress response to the conjugates, cannot be distinguished on the basis of these data.

The upregulation of multiple c-di-GMP phosphodiesterases alongside downregulation of a cyclase appears counterintuitive given the established role of elevated c-di-GMP in promoting biofilm formation [45]. This pattern may reflect high-flux c-di-GMP cycling rather than net depletion, or a later maturation phase in which second messenger levels are reduced to permit structural reorganization of the developing biofilm [46]. The 4-h sampling time point captures an early stage of the response, and time-course measurements would be needed to fully resolve these dynamics.

The induction of polysaccharide deacetylases is consistent with modification of the cell surface, but does not by itself demonstrate altered extracellular-polysaccharide chemistry or stabilization of the structures visualized by microscopy (Fig. 3) [47].

The induction of the RelE/ParE toxin-antitoxin system could provide a molecular basis for the reduced apparent planktonic growth rates observed after polymer addition (Fig. 2) [48]. RelE-family toxins inhibit translation by mRNA cleavage at the ribosome, generating slow-growing persister cells that are characteristic of biofilm communities [49], [50], [51]. However, this signature is not specific to biofilm formation and can also be compatible with translation arrest and persistence in planktonic cultures. The massive downregulation of catabolic gene clusters—BCAA degradation, pyruvate and alpha-ketoacid dehydrogenase complexes, ethanolamine utilization, and carbon monoxide dehydrogenase—is consistent with the reduced biomass-specific metabolic activity that is a hallmark of biofilm physiology but would be equally fitting to growth repressed cells [52].

The core regulatory response was shared across treatments and catabolic repression was broadly shared across treatments, with pathway-specific differences (strongest under C and B for branched-chain amino-acid and acetyl-CoA-synthase genes; comparable under K for pyruvate and CO dehydrogenase). Hexanal (K) showed the highest cytotoxicity in the live/dead analysis, consistent with its known membrane-damaging properties [36].

As the transcriptomic and viability analyses were performed under heterotrophic screening conditions, the extent to which the observed transcriptional and physiological responses are representative of cells under electroautotrophic, CO_2_-fixing conditions remains to be confirmed. We further note that the screening assays did not include a fully cell-free abiotic control carried through the complete crystal-violet protocol for each conjugate, nor an independent cell-specific biomass measurement; the screening read-outs should therefore be regarded as exploratory, relative indicators rather than absolute quantifications of cellular biomass.

The addition of the polymer to the bioelectrochemical system had a major influence on the thickness of the cathodic layer. Still, a central limitation of this experiment is that growth kinetics and number of entrapped cells versus cells growing unentrapped cannot be resolved. With this caveat, addition of the polymer-2-naphthaldehyde conjugate led to a faster increase and greater maximum height of the cathode-associated layer than in the biotic control. For actual application, particularly in the food sector, further investigation would be necessary to determine how well the biomass can be separated from the polymer. Nevertheless, accelerated cathode coverage by polymer-aldehyde conjugates may represent a starting point for addressing the start-up phase limitation of microbial electrosynthesis. Whether this translates into improved process efficiency, as opposed to, for example, impeded mass transfer or reduced electrode access for active cells, remains to be established through dedicated electrochemical characterization.

The calculations of the mean height showed that the increase was remarkably faster when the polymer was added to the cell culture compared to the biotic negative control. Moreover, the maximum height of the cathodic layer was greater.

## 5 Conclusion and future prospective

This study indicates that incubation with polymer-aldehyde conjugates can promote cell aggregation by *K. spormannii* EA-1. Faster cathode coverage is hypothesized to reduce the abiotic electron losses characteristic of the oMES start-up phase; however, this remains to be tested directly, and faster coverage by a polymer-rich layer could equally impede mass transfer or reduce access of active cells to the electrode.

Among the twelve polymer-aldehyde conjugates screened, functionalization with 2-naphthaldehyde emerged as the most promising candidate, increasing the maximum OCT-measured layer height more than sixfold (>1 mm vs. 0.17 mm in the biotic control) within seven days. As the abiotic polymer-only control also formed a substantial layer (0.24 mm), this height increase reflects the combined contribution of polymer deposition and cell aggregation rather than viable biofilm biomass alone. Its functional relevance for electron transfer and process performance remains to be established. The mechanism appears to involve polymer-mediated aggregation of bacterial cells, offering a potential route to accelerate cathode coverage without genetic modification. The combined presence of cells and polymer produced a substantially thicker cathode-associated layer than either component alone, indicating a synergistic contribution of cellular aggregation and polymer deposition to layer formation. This is particularly valuable given the lack of established genetic tools for this organism. Because the reactor experiment was performed with a single biological reactor per condition, these findings should be regarded as a qualitative proof-of-concept rather than a statistically validated result.

Future work should clarify whether active cell growth continues after polymer-mediated entrapment, optimize polymer concentrations, and develop strategies for biomass-polymer separation in food/feed applications. Cell-specific viability, metabolic activity, and electrochemical performance (e.g., current density, coulombic efficiency) within the reactor; including direct viability assessment of the cathode-associated layer after reactor operation, were not assessed in this study and represent a priority for future work.

Despite these open questions, pAcU-aldehyde conjugates represent a promising starting point for addressing start-up phase limitations in oMES technology, warranting further mechanistic and functional validation toward sustainable bioelectrochemical production of biomass and polyhydroxyalkanoates from CO_2_ and renewable electricity.

## CRediT authorship contribution statement

**Leonie Rominger:** Writing – original draft, Visualization, Validation, Methodology, Investigation. **Amit Deb:** Writing – original draft, Methodology, Investigation. **Christian Jonas Lapp:** Visualization, Formal analysis, Data curation. **Francisco (Paco) Fernandez-Trillo:** Writing – original draft, Supervision, Funding acquisition, Conceptualization. **Johannes Gescher:** Writing – original draft, Supervision, Funding acquisition, Conceptualization.

## Declaration of competing interest

The authors declare that they have no known competing financial interests or personal relationships that could have appeared to influence the work reported in this paper.

## Data Availability

Data will be made available on request.The raw sequencing data generated during this study have been deposited in the NCBI Sequence Read Archive (SRA) under accession number PRJNA1505563.
