# Supplementary material for: Synthetic polymers promote cell aggregation and accelerate cathode-associated layer formation in Kyrpidia spormannii EA-1
Source: Biofilm. 2026 Sep 15;12:100399. doi: 10.1016/j.bioflm.2026.100399 (PMC13602148; doi:10.1016/j.bioflm.2026.100399)
Supplement: Supplementary file 1 [file mmc1.docx]

# Supplementary Information

**Figure S1.** Growth curves of cell cultures treated with polymer-aldehyde conjugates. (A) Cell cultures were incubated with polymer-aldehyde conjugates (B, C, I, or K). (B) Control cultures received aldehyde only, untreated polymer (pAcU) (CP), solvents only (CS), or no treatment (CZ). Optical density was measured at 600 nm over 95 h. Data represent mean ± standard deviation (n = 3).

**Figure S2.** Principal component analysis of transcriptomic data. Principal components 1 and 2 account for 70% of the variance in the data sets. Shown are the duplicates of the transcriptome analysis for cell cultures treated with polymer-aldehyde conjugates (B, C, or K), and the untreated reference culture.

**Figure S3**.^1^H-NMR spectra of *tert*-butyl 2-(1H-imidazole-1-carbonyl)hydrazine-1-carboxylate.

**Figure S4**. C^13^-NMR spectra of *tert*-butyl 2-(1H-imidazole-1-carbonyl)hydrazine-1-carboxylate.

**Figure S5.** ^1^H-NMR spectra of N-propargyl-N’-(*tert*-butoxycarbonyl)amino urea.

**Figure S6.** C^13^-NMR spectra of N-propargyl-N’-(*tert*-butoxycarbonyl)amino urea.

**Figure S7**. ^1^H-NMR spectra of poly(N-propargyl-N’-(*tert*-butoxycarbonyl)amino urea)

(pAcU-Boc).

**Figure S8.** ^1^H-NMR spectra of poly(N-propargyl-N’-amino urea) (pAcU).

**[Table S1 is an additional file]**

**Table S1.** Gene expression data by transcriptome analysis. Fold-change values are shown for each aldehyde treatment relative to the reference.
